# The Comparative Nutrient Value of Meat Preparations

**Published:** 1896-05-16

**Authors:** R. Hill Shaw


### THE COMPARATIVE NUTRIENT VALUE OF MEAT PREPARATIONS.

MR. R. HILL SHAW writes from Llansilin: In referring to condensed foods on page 26 of your issue of April 11th, you allude to a recent article in *Food and Sanitation* "on the comparative worthlessness of many of the highly recommended meat extracts and condensed food preparations," and in the case "of one of these much-praised meat juices," namely, Valentine's, you quote their opinion that, "as far as nutrient value goes, the water in which dinner plates are washed would be about as valuable." May I point out to you that, with regard to this statement, the *Lancet* (March 7th) says: "We, on the other hand, know this opinion to be rubbish, and have challenged our contemporary to appoint with our approval an analyst of well-known repute to undertake an independent analysis, and to publish it side by side with the analysis upon which this opinion is based. No notice has been taken of our challenge." Again, with respect to Bovril, *Food and Sanitation* (pinning their faith on an analysis by Professor Chittenden, of Yale University, in 1891) pointed out about the same time that the value of this article as a nutrient was about seventeen times greater than that of Valentine's Meat Juice; whilst the *Lancet*, on the other hand, asserts (February 22nd) that Bovril is "not comparable" to Valentine's Meat Juice. As doubtless the views of the *Lancet* on these points has escaped your notice, I would ask you to lay them before your readers so that they may have an opportunity of forming their own conclusions as to the relative merits of these two foods. The whole discussion which has lately taken place in the two papers named above with reference to meat preparations in general is very interesting and well worthy of a perusal, though I cannot see why it has been left in its present unsettled condition. Considering the very serious importance both to the sick and to the medical profession, which a true understanding in the matter represents, I think both journals ought to bury the hatchet, and, for the public good, they should submit their differences to an independent inquiry, as suggested by the *Lancet*. This I still hope to see by the paper challenged picking up the gauntlet which has been thrown down to it. If in the right it has everything to gain by such an investigation.

